# Clinical and economic outcomes of remotely delivered cognitive behaviour therapy versus treatment as usual for repeat unscheduled care users with severe health anxiety: a multicentre randomised controlled trial

**DOI:** 10.1186/s12916-019-1253-5

**Published:** 2019-01-23

**Authors:** Richard Morriss, Shireen Patel, Sam Malins, Boliang Guo, Fred Higton, Marilyn James, Mengjun Wu, Paula Brown, Naomi Boycott, Catherine Kaylor-Hughes, Martin Morris, Emma Rowley, Jayne Simpson, David Smart, Michelle Stubley, Joe Kai, Helen Tyrer

**Affiliations:** 1 Division of Psychiatry and Applied Psychology, Institute of Mental Health, University of Nottingham, Triumph Road, Nottingham, NG7 2TU UK; 2 Division of Rehabilitation and Ageing, University of Nottingham, School of Medicine, Nottingham, NG7 2UH UK; 3 Nottinghamshire Healthcare NHS Foundation Trust, Duncan MacMillan House, Porchester Road, Nottingham, NG3 6AA UK; 4 Leicestershire Partnership NHS Trust, Plaza, Riverside House Bridge Park, Bridge Park Road, Thurmaston, Leicester, LE4 8PQ UK; 5 Business School, University of Nottingham, Wollaton Road, Nottingham, NG8 1BB UK; 6 Leicester Terrace Health Centre, Adelaide St, Northampton, NN2 6AL UK; 7 Division of Primary Care, University of Nottingham, School of Medicine, Nottingham, NG7 2UH UK; 8 Department of Psychiatry, Imperial College, South Kensington Campus, London, SW7 2AZ UK

**Keywords:** Illness anxiety disorder, Hypochondriasis, Depression, High care costs, Urgent care, Family care, Cognitive behaviour therapy, Digital, Videoconferencing, Remote therapy

## Abstract

**Background:**

It is challenging to engage repeat users of unscheduled healthcare with severe health anxiety in psychological help and high service costs are incurred. We investigated whether clinical and economic outcomes were improved by offering remote cognitive behaviour therapy (RCBT) using videoconferencing or telephone compared to treatment as usual (TAU).

**Methods:**

A single-blind, parallel group, multicentre randomised controlled trial was undertaken in primary and general hospital care. Participants were aged ≥18 years with ≥2 unscheduled healthcare contacts within 12 months and scored >18 on the Health Anxiety Inventory. Randomisation to RCBT or TAU was stratified by site, with allocation conveyed to a trial administrator, research assessors masked to outcome. Data were collected at baseline, 3, 6, 9 and 12 months. The primary outcome was change in HAI score from baseline to six months on an intention-to-treat basis. Secondary outcomes were generalised anxiety, depression, physical symptoms, function and overall health. Health economics analysis was conducted from a health service and societal perspective.

**Results:**

Of the 524 patients who were referred and assessed for trial eligibility, 470 were eligible and 156 (33%) were recruited; 78 were randomised to TAU and 78 to RCBT. Compared to TAU, RCBT significantly reduced health anxiety at six months, maintained to 9 and 12 months (mean change difference HAI –2.81; 95% CI –5.11 to –0.50; *P* = 0.017). Generalised anxiety, depression and overall health was significantly improved at 12 months, but there was no significant change in physical symptoms or function. RCBT was strictly dominant with a net monetary benefit of £3,164 per participant at a willingness to pay threshold of £30,000. No treatment-related adverse events were reported in either group.

**Conclusions:**

RCBT may reduce health anxiety, general anxiety and depression and improve overall health, with considerable reductions in health and informal care costs in repeat users of unscheduled care with severe health anxiety who have previously been difficult to engage in psychological treatment. RCBT may be an easy-to-implement intervention to improve clinical outcome and save costs in one group of repeat users of unscheduled care.

**Trial registration:**

The trial was registered at ClinicalTrials.gov on 19 Nov 2014 with reference number NCT02298036

**Electronic supplementary material:**

The online version of this article (10.1186/s12916-019-1253-5) contains supplementary material, which is available to authorized users.

## Introduction

In health services, unscheduled same-day care is defined as any unplanned contact with a health service by a person requiring or seeking help, care or advice [1]. Globally, unscheduled same-day care is increasing across primary and hospital care settings, presenting a burgeoning challenge for health systems [2–4]. Severe health anxiety, which is defined as a preoccupation with health worries or a belief that one might have a serious physical illness [5], is one possible reason for repeated use of unscheduled care. Severe health anxiety is a common feature of healthcare, with a lifetime prevalence of 8.5% in primary care and 24% in hospital clinics [6, 7]. Severe health anxiety can lead to increased unscheduled care use for reassurance and increased medical investigations, or delayed healthcare attendance followed by catastrophic emergency presentation because of anxiety-related healthcare avoidance. It may increase people’s functional impairment, sickness absence, risk of cardiovascular disease and other chronic physical or mental health problems and increase healthcare costs [8–10].

Cognitive behaviour therapy (CBT) is a well-evidenced treatment for severe health anxiety, with benefits lasting at least five years [11–13]. Despite the effectiveness of CBT for health anxiety, uptake is typically low because of stigma and negative experiences of mental health services [14, 15]. Remotely delivered CBT (RCBT), whereby patients talk directly to a CBT therapist using internet-based videoconferencing and/or the telephone, may enhance accessibility, acceptability and service capacity for patients with severe health anxiety. For anxiety and somatic disorders, RCBT can achieve comparable outcomes to face-to-face treatment [16, 17]. Digitally delivered psychological therapies are generally more effective when supported by a therapist, as opposed to being unguided or self-guided [18, 19]. Therefore, use of a therapist via the phone or online might maintain the efficacy of CBT for health anxiety. Furthermore, remote delivery may improve access for those who are concerned about using mental health services, live in rural areas with reduced access to services, travel frequently or are restricted by comorbid health conditions, family care or work commitments.

The rationale for this study was that if repeat users of unscheduled care with severe health anxiety could be easily identified in routine healthcare settings by health professionals with whom they already have an established relationship, then an acceptable explanation of their problems might be facilitated through a trusted source. Offering RCBT in an accessible format, via videoconferencing or telephone, may then enable participants to become engaged with treatment more easily. We used a model of CBT that previously showed sustained reductions in health anxiety over five years [11], but delivered it remotely to a targeted high service-use population. For health anxiety, CBT promotes self-management techniques that may sustainably improve health anxiety. This may reduce the need to seek reassurance from health professionals and informal carers, or more timely care for serious health problems may be sought, reducing the need for emergency in-patient care. In turn, health and informal care costs might be reduced.

The primary aim of this study was to assess the clinical and economic outcomes of RCBT via videoconferencing or telephone to repeat users of unscheduled care with severe health anxiety compared to usual care.

## Methods

### Study design and participants

We conducted a single-blind, patient-level, parallel group, multicentre randomised controlled trial (RCT) in primary and secondary care centres across the East Midlands and at two other English sites. Participants were recruited from emergency departments, walk-in centres, hospital outpatient clinics and GP practices offering same-day appointments. Participants were approached if they were aged 18 years and over; had two or more unscheduled consultations with any healthcare provider in the last 12 months, not attributed to identified pathology; met criteria for clinical severity of health anxiety (a score of 18 or above on the 14-item Short Health Anxiety Inventory, SHAI [20, 21]) and had sufficient understanding of English to engage with the intervention. A SHAI score ≥18 is the clinical cut-off used by Improving Access to Psychological Therapies in the UK to identify patients who meet the diagnostic criteria for hypochondriasis/severe health anxiety. Patients were excluded if they were at immediate risk of harm to themselves or others; had moderate to severe learning disability; a serious mental or physical illness, including substance use disorder, to the extent that engagement in the intervention would not be possible, e.g. communication difficulties; were receiving ongoing investigations for a pathological medical condition; or had received specialist mental health intervention within the previous six months. We intentionally included those diagnosed with chronic physical conditions or common mental health problems. The trial protocol is published [22].

### Randomisation and masking

Randomisation was determined by a computer-generated pseudo-random code using random permuted blocks of 2, 4 or 6, created by the Nottingham Clinical Trials Unit in accordance with their standard operating procedure and held on a secure server. Participants were allocated with equal probability (1:1) to each treatment arm, with stratification by site. The administration officer relayed treatment allocation information to CBT therapists and participants. Only the trial manager and the administration support officer had password access to the un-blinded randomisation data. The researchers responsible for collecting the baseline and outcome data were blind to randomisation until collection of all data was complete. Any instances of un-blinding were recorded.

### Procedures

The principles of user-design and co-production were utilised by developing a network of patient and public involvement (PPI) members, health practitioners and researchers contributing at all stages of the study including study design, recruitment, RCBT delivery methods and interpretation of results [23]. In particular, iterative feedback with this network of practice and the experience of PPI members meant we could develop and use engaging, non-stigmatising explanations in study procedures and materials and adopt simple, flexible procedures to identify, recruit and retain patients.

Referring clinicians approached patients meeting eligibility criteria and sought consent for them to be contacted by study researchers. Referrers explained to patients that the aim of the study was to find out whether talking to a health professional via videoconferencing or telephone might help them to cope better with distress linked to their bodily symptoms. Most practitioners approached patients opportunistically during usual care consultations, based on their clinical knowledge of the patient. They also used electronic administrative prompts on patient medical notes, which highlighted repeat users of urgent ‘same-day’ appointments. Some practitioners posted study invitation letters to patients who might meet the study criteria after electronically searching patient records. One primary care centre sent out text messages to such patients.

A researcher telephoned potential participants who provided written or verbal consent. The researcher explained they had been referred to the study because they had been experiencing physical symptoms and associated distress and that talking therapy via videoconferencing or telephone may help to manage distress. Participants were informed they had a 1 in 2 chance of receiving the RCBT intervention and that the study was being offered as an additional source of support, rather than replacing or restricting their current healthcare use. No participant was told that an aim of the study was to reduce service use. Further study information was posted to all participants prior to a baseline assessment interview, which was arranged at a time and place suitable for the patient. Verbal and written consent was received at this baseline assessment interview. Following assessment and confirmation of eligibility, participants were randomly allocated to one of two treatment arms: RCBT in addition to usual treatment, or treatment as usual (TAU).

#### Remote cognitive behaviour therapy (RCBT)

Participants allocated to the RCBT arm were provided with a detailed information sheet about RCBT, an RCBT guide, a manual to help set up the videoconferencing system (where used) and a contingency management plan for when connection was poor or not possible. A CBT therapist contacted participants within 10 days of randomisation. A team of four experienced CBT therapists remotely delivered CBT for health anxiety using a treatment manual developed from the Cognitive Behavioural Therapy for Health Anxiety in Medical Patients (CHAMP) study [15]. Between six and 12 sessions of CBT were offered, with up to three booster sessions if required. This included an initial ‘setup’ session, during which the methods used to adapt CBT to remote delivery were discussed and any concerns about this method were addressed. The number of sessions offered depended on treatment response: more sessions were offered when treatment response was slower. Treatment used CBT principles to assess and test beliefs about health, illness and associated issues that were likely to be causing distress. Behavioural strategies known to maintain health anxiety were identified and collaboratively reduced or stopped (Additional file 1: Table S1). Participants also received text messages/email reminders about their CBT sessions. Participants were free to continue to consult their usual healthcare providers other than the CBT therapist throughout the intervention and after treatment completion.

RCBT was delivered via a videoconferencing system called WebEx or by telephone, depending on the participant’s preference. WebEx was selected because of connection security and interactive utilities enabling an experience close to face-to-face CBT. At the time of assessment, compared to nine other piloted systems, WebEx was also comparatively cheaper. Permission was sought to record audio/video during treatment sessions. These were accessible to participants as a means of consolidating learning from each session.

Recordings were also used for assessment and improvement of therapeutic quality in the clinical supervision of CBT therapists. Group clinical supervision was conducted monthly via WebEx. Audio/video recordings of the most recent sessions with patients who were not responding to treatment were reviewed and appropriate treatment approaches were discussed. The lead therapist from the CHAMP trial (HT) conducted clinical oversight [11]. A version of the Cognitive Therapy Rating Scale Revised (CTSR) [24], adapted to health anxiety, was used for two randomly selected sessions given by each therapist at four time points to assess, maintain and improve therapist competence and treatment fidelity. In this way, remote delivery meant that a few therapists could be used to consistently deliver the intervention over a large geographical area.

#### Treatment as usual (TAU)

Usual care included informing the participant, their GP and any other healthcare referrer of the participant’s involvement in the study. For participants whose baseline assessment scores suggested risk of harm to themselves or others, or if they required a referral to a different service, a phone call was made and a letter sent to referrers including to deliver information regarding the patient’s severity of health anxiety, risk of harm, other comorbidities and their quality of life. Treatment as usual was unconstrained.

### Outcomes

The primary clinical outcome was change in SHAI score from baseline to six months. Secondary outcomes were change in the following self-reported measures from baseline to 12 months:

- SHAI [21].
- 7-item Generalised Anxiety Disorder for anxiety (GAD-7) [25].
- 15-item Patient Health Questionnaire for somatic distress (PHQ-15) [26].
- 9-item Patient Health Questionnaire for depression (PHQ-9) [27].
- 8-item Work and Social Adjustment Scale for social function (WSAS) [28].
- 5-item quality of life on the EQ5D-5L including the Visual Analogue Scale (VAS) [29].
- 36-item Short Form Health Survey (SF-36) [30].
- Change in health care service utilisation established through an adapted Client Service Receipt Inventory (CSRI) [31].

At the baseline assessment only, participants were interviewed using a standardised psychiatric interview [32] to identify mental disorders. All baseline assessments were conducted by te study researchers, either face-to-face or by telephone, dependent on the participant’s preference. All measures were selected because they have a history of routine use in settings within the UK National Health Service (NHS), should RCBT be implemented into NHS practice. Follow-up assessments were carried out at three, six, nine and 12 months by telephone, email, videoconferencing, post, or face-to-face, dependent on participant preference.

### Health economics

Health economic analysis was conducted from a health service and societal perspective. Resource use data were collected at baseline, three, six, nine and 12 months using the adapted CSRI, which included sections on outpatient hospital appointments, inpatient stays, primary and community care services, medicines and informal care visits. For each type of health and social care services use, an appropriate unit cost was identified and valued using NHS reference costs from the reference year 2017 [33] for both the RCBT intervention and TAU groups. The intervention cost of using the technology was further determined. Outcome was obtained in the same periods using the EQ5D-5L [29] to calculate quality-adjusted life-years (QALYs).

The incremental cost-effectiveness ratio (ICER) was calculated for the RCBT intervention versus treatment as usual:

$$ \mathsf{ICER}=\left({\mathsf{Cost}}_{\mathsf{CBT}}-{\mathsf{Cost}}_{\mathsf{TAU}}\right)/\left({\mathsf{QALY}}_{\mathsf{CBT}}-{\mathsf{QALY}}_{\mathsf{TAU}}\right) $$

The threshold of £30,000 per QALY, set by the National Institute for Health and Care Excellence (NICE) [34] to assess the cost-effectiveness of RCBT. The RCBT intervention is deemed to be cost-effective if ICERs fall below this value.

### Statistical analysis

The analysis was conducted on an intention-to-treat basis. Multilevel modelling was performed to quantify the treatment effects, with patient as a level 2 unit. Time, treatment status and treatment × time interactions and baseline measurements were included as covariates. Secondary outcomes were analysed in a similar way. Missing values were explored and imputed under the Missing At Random assumption [35]. Sensitivity analysis was conducted with the same multilevel model on observed data to check the result’s robustness to missingness. Stata 15 (StataCorp LLC, USA) and Realcom-impute (University of Bristol, UK) software were used for data analysis. More details can be found on the trial statistical analysis plan [36].

#### Sample size and calculation

The results of the CHAMP study [15] showed that the mean SHAI scores for CBT and TAU groups were 24.9 (SD 4.2) and 25.1 (SD 4.5), respectively, at baseline and 17.7 (SD 8.0) and 22.6 (SD 6.8), respectively, at six months. Therefore, 114 participants were required to detect such a difference in SHAI score at six months to achieve 90% power at a two-tailed significance level of 0.05, assuming equal SD (8.0) for both groups and null correlation between baseline and follow-up measures for the purpose of being conservative. After taking into account a 20% loss to follow-up rate, a required sample size of 144 was calculated. Stata 14 (StataCorp LLC, USA) was used to run the power analysis. In September 2016, we observed that our follow-up rate for the primary outcome measure was 75%. Thus, to detect a difference, the sample size was increased to 152. Approval was obtained from the study sponsor, an independent scientific committee and ethics committee to recruit additional participants, resulting in a sample size of 156 participants.

## Results

**Fig. 1:**
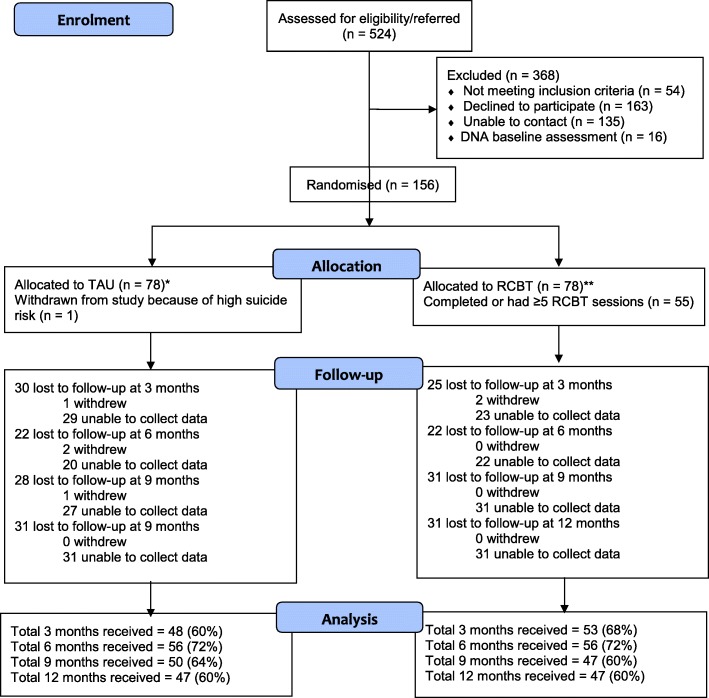
Consort diagram: participant flow into randomised controlled trial. *There was one randomization protocol violation. One participant who was allocated into TAU by the randomisation system was accidentally sent the incorrect treatment allocation letter, resulting in them receiving the RCBT therapy. This error was identified following the completion of treatment. The participant completed outcome data only at three months. **There was an enrolment protocol violation. Two participants in the RCBT group did not meet the criteria of ≥18 on the SHAI. This error was not identified until final analysis and as such both participants were included in the analysis

Of the 524 patients referred to the study and assessed for eligibility between 19 November 2014 and 31 December 2016, 470 were eligible and 156 (33%) participants were recruited. Seventy-eight participants were allocated to RCBT and 78 to TAU (Fig. 1). Researchers were inadvertently un-blinded to 17 participants in the RCBT group and one participant in the TAU group. No treatment-related adverse events were reported in either group.

Baseline characteristics for the two groups were similar (Table 1 and Additional file 1: Table S2). At baseline, RCBT and TAU groups had median unscheduled face-to-face healthcare consultations of 6.5 (IQR 3-12) and 5 (IQR 3-10), respectively, in the preceding 12 months. Most participants (80%) were referred from primary care by their GP. Two-thirds were female and half were under 35 years of age, although there was a wide age range. At baseline, mean (SD) SHAI scores for the RCBT and TAU arms were 27.31 (5.38) and 26.41 (5.13), respectively. Table 1 shows that the mean (SD) scores at baseline on the GAD-7 and the PHQ-9 were above clinical cut-offs ( >8 and>10, respectively) for both groups [21], indicating that clinically important comorbid generalised anxiety and depression were typically present alongside severe health anxiety. There were no clinically important differences at baseline for any demographic or clinical characteristic between the two treatment arms.

**Table 1:** Baseline characteristics of participants

	RCBT (*n* = 78)	TAU (*n* = 78)
Age, median (range) years	31.12 (18.36–79.10)	33.28 (18.69–82.71)
Female, n (%)	56 (72)	52 (67)
Occupational status		
Employed, n (%)	32 (41)	29 (37)
Student/training, n (%)	27 (35)	24 (31)
Homemaker, n (%)	0	2 (3)
Retired, n (%)	7 (9)	10 (13)
Unemployed, n (%)	12 (15)	13 (17)
Highest qualification		
First degree or higher, n (%)	24 (31)	25 (32)
A-Level or other higher qualification, n (%)	28 (36)	30 (38)
O-Level/GCSE or other qualification, n (%)	19 (24)	20 (26)
No qualifications, n (%)	7 (9)	3 (4)
Marital status		
Married/partner, n (%)	32 (41)	39 (50)
Single, n (%)	34 (45)	32 (41)
Divorced/widowed, n (%)	10 (13)	7 (9)
Ethnicity		
White British, n (%)^a^	58 (74)	58 (74)
Referral type		
GP, n (%)	63 (81)	62 (79)
Medical specialties, n (%)	9 (12)	7 (9)
Emergency department, n (%)	4 (5)	6 (8)
Walk-in centre, n (%)	2 (3)	3 (4)
Baseline scores		
SHAI, mean (SD)	27.31 (5.38)	26.41 (5.13)
GAD7, mean (SD)	12.94 (5.49)	12.68 (6.13)
PHQ9, mean (SD)	13.35 (6.50)	13.12 (6.71)
PHQ15, mean (SD)	14.16 (5.63)	13.90 (6.45)
EQ5D-5L Utility Value, mean (SD)	0.61 (0.28)	0.60 (0.29)
VAS, mean (SD)	54.76 (21.72)	56.97 (22.58)
WSAS, mean (SD)	19.33 (11.40)	20.35 (10.54)
SF36 Physical functioning, mean (SD)	67.29 (30.39)	64.18 (32.04)
Role limitations – physical health, mean (SD)	29.81 (39.08)	28.90 (40.77)
Role limitations – emotional problems, mean (SD)	35.47 (42.07)	27.35 (41.17)
Energy/fatigue, mean (SD)	25.73 (20.39)	27.05 (22.48)
Emotional well-being, mean (SD)	40.92 (19.88)	42.54 (24.83)
Social functioning, mean (SD)	45.83 (29.10)	46.31 (30.82)
Pain, mean (SD)	47.47 (24.83)	41.38 (26.96)
General health, mean (SD)	28.27 (19.29)	36.25 (22.89)
Unscheduled care attendances in last 12 months, median (range)	6.5 (2.0–125.0)	5.0 (2.0–34.0)

^a^further detail provided in Additional file 1

RCBT participants received a median of eight sessions (range: 0–16) with 55 (71%) participants completing therapy or attending ≥5 sessions. Forty-seven (60%) received sessions via WebEx, 20 (25%) had sessions by telephone and 11 (14%) received no sessions. Therapist competency assessments using the CTSR indicated CBT skill levels between the ‘competent’ and ‘proficient’ range for all therapists, with an overall trend of improved skill over time. Follow-up completion rates were similar between the two groups. In TAU and RCBT arms, 8 (10.2%) and 2 (2.6%, 7–12 months) participants, respectively, received ≥2 sessions of any additional psychological treatment.

**Fig. 2:**
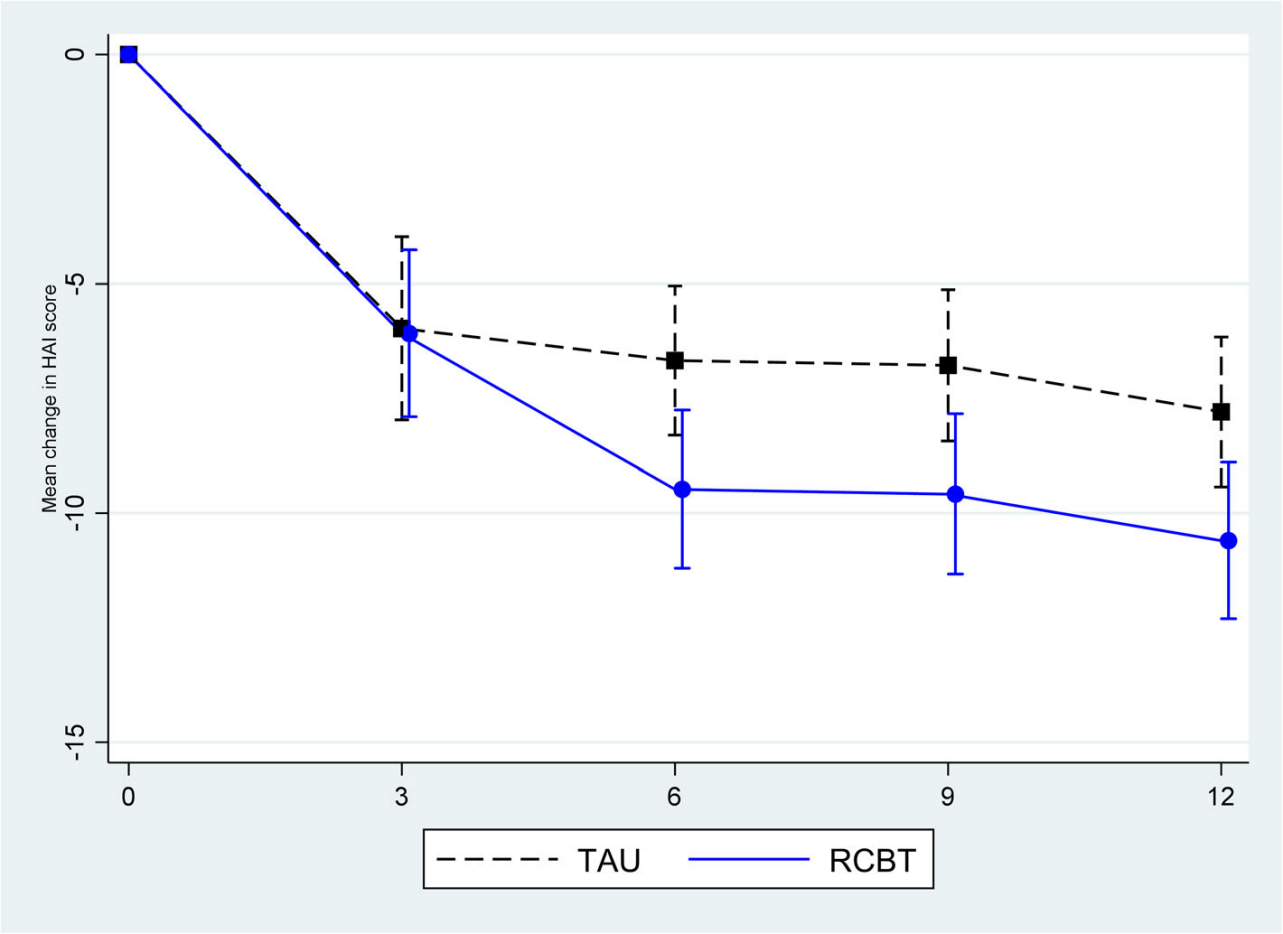
Mean (95% CI) change in 14-item Short Health Anxiety Inventory over 12 months remote cognitive behaviour therapy versus treatment as usual

Table 2 and Fig. 2 show that participants in both arms experienced a reduction in health anxiety, with participants in the RCBT arm showing significantly greater reduction at six months (mean change difference at six months –2.81 95% CI –5.11 to –0.50; *P* = 0.017), 9 and 12 months. Analysis confined only to participants with complete data at baseline and six months showed nearly identical results (Additional file 1: Figure S1 and Table 3). Recovery rates on the SHAI are shown in Additional file 1: Table S4. Participants in the RCBT arm also showed significantly greater reductions in generalised anxiety (GAD-7) at six and 12 months, depression (PHQ-9) at 12 months and overall health (VAS) at 12 months. No significant differences were found on the PHQ-15 or WSAS between the two groups at any time point. SF-36 results confirm improvements in general health, with no differences in any other domain (Additional file 1: Table S5).

**Table 2:** Multilevel modelling of changes in score from baseline to 12 month follow-up for remote cognitive behavioural therapy (RCBT) intervention versus treatment as usual (TAU)

	RCBT		TAU		Comparison	
	N	Mean change from baseline (95% CI)	N	Mean change from baseline (95% CI)	Difference (95% CI)	*P*-value
SHAI						
3 months	53	–6.08(–7.90 to –4.26)	48	–5.97(–7.97 to –3.97)	–0.11(–2.82 to 2.60)	0.936
6 months	56	–9.48(–11.20 to –7.75)	56	–6.67(–8.30 to –5.04)	–2.81(–5.11 to –0.50)	0.017
9 months	46	–9.58(–11.33 to –7.84)	50	–6.78(–8.43 to –5.12)	–2.81(–5.11 to –0.50)	0.017
12 months	47	–10.60(–12.31 to –8.89)	47	–7.79(–9.43 to –6.16)	–2.81(–5.11 to –0.50)	0.017
GAD 7						
3 months	52	–3.69(–5.09 to –2.28)	48	–4.79(–6.17 to –3.41)	1.10(–0.84 to 3.05)	0.265
6 months	53	–6.22(–7.62 to –4.82)	55	–3.83(–5.21 to –2.44)	–2.39(–4.40 to –0.39)	0.020
9 months	43	–5.37(–7.05 to –3.69)	48	–4.48(–5.97 to –2.99)	–0.89(–3.00 to 1.23)	0.408
12 months	45	–6.68(–8.14 to –5.21)	46	–3.92(–5.40 to –2.45)	–2.75(–4.82 to –0.68)	0.009
PHQ9						
3 months	52	–3.24(–4.69 to –1.80)	47	–3.01(–4.38 to –1.64)	–0.23(–2.15 to 1.68)	0.812
6 months	53	–4.67(–6.02 to –3.33)	55	–3.11(–4.46 to –1.76)	1.56(–3.45 to 0.33)	0.105
9 months	43	–4.41(–6.06 to –2.76)	48	–3.24(–4.78 to –1.69)	–1.17(–3.18 to 0.83)	0.250
12 months	45	–5.05(–6.37 to –3.72	45	–2.69(–4.06 to –1.31)	–2.36(–4.22 to –0.50)	0.013
PHQ15						
3 months	52	–0.35(–1.58 to 0.88)	48	–1.68(–3.16 to –0.19)	1.33(–0.52 to 3.17)	0.157
6 months	54	–2.95(–4.11 to –1.78)	55	–1.62(–2.90 to –0.34)	–1.33(–3.09 to 0.42)	0.137
9 months	45	–2.62(–4.19 to –1.04)	49	–2.20(–3.47 to –0.92)	–0.42(–2.38 to 1.53)	0.669
12 months	46	–3.33(–4.62 to –2.04)	47	–1.77(–2.99 to –0.55)	–1.57(–3.37 to 0.24)	0.089
EQ5D–5L (VAS)						
3 months	52	8.74 (3.70 to 13.79)	47	2.80(–2.35 to 7.96)	5.94(–1.22 to 13.11)	0.103
6 months	54	13.66 (8.97 to 18.34)	55	11.10 (6.32 to 15.89)	2.56(–4.03 to 9.14)	0.446
9 months	45	10.24 (5.28 to 15.21)	49	2.92 (–2.06 to 7.90)	7.33(–0.02 to 14.67)	0.051
12 months	46	13.80 (8.90 to 18.71)	47	4.25 (–0.87 to 9.37)	9.56 (2.73 to 16.39)	0.006
WSAS						
3 months	51	–4.45(–6.63 to –2.27)	48	–3.36(–5.61 to –1.10)	–1.10(–4.07 to 1.87)	0.468
6 months	53	–7.52(–9.85 to –5.19)	55	–4.89(–7.03 to –2.75)	–2.63(–5.72 to 0.45)	0.094
9 months	44	–7.31(–9.81 to –4.81)	56	–5.77(–8.15 to –3.39)	–1.54(–4.99 to 1.90)	0.377
12 months	45	–7.89(–10.22 to –5.56)	56	–5.40(–7.79 to –3.01)	–2.49(–5.73 to 0.75)	0.131
	RCBT		TAU		Unadjusted difference in change	
	*N*	Mean (SD)	*n*	Mean (SD)	Mean (95% CI) (*P*-value)	
QALYs^a^ at 12 months	31	0.66 (0.22)	25	0.59 (0.31)	0.07 (0.07 to 0.21) (0.332)	

^a^QALYs at 12 months is based on the complete dataset over the 12-month follow-ups

**Table 3:** Costs (£) per participant over 12-month follow-up period remote cognitive behaviour therapy versus treatment as usual

	RCBT		TAU		Unadjusted difference in change
	*n*	Mean (SD)	*n*	Mean (SD)	Mean (95% CI) (*P*-value)
CBT sessions	78	496 (294)	78	0 (0)	496 (431 to 562) (0.000)
Technology	78	35.8 (0)	78	0 (0)	35.8 (35.8 to 35.8) (N/A.)
Outpatient hospital visits	35	608 (871)	34	728 (706)	–120 (–502 to 261) (0.531)
Inpatient hospital visits	38	22 (87)	37	258 (656)	–236 (–450 to –23) (0.031)
Primary and community care	38	798 (838)	35	2066 (5224)	–1,268 (–2,981 to 444) (0.144)
Travel	33	36 (46)	35	50 (109)	–14 (–55 to 27) (0.495)
Medication	37	219 (402)	34	436 (1082)	–217 (–597 to 163) (0.259)
Informal care	36	162 (536)	34	293 (1253)	–130 (–586 to 325) (0.569)
Total cost	29	2197 (1048)	26	3261 (5010)	–1064 (–2973 to 845) (0.269)

**Table 4:** The net monetary benefit of remote cognitive behaviour therapy versus treatment as usual at various willingness to pay (WTP) thresholds using EQ-5D-5L utilities

Net monetary benefit							
WTP threshold	£5,000	£10,000	£15,000	£20,000	£25,000	£30,000	£35,000
Observed values	£1,414	£1,764	£2,114	£2,464	£2,814	£3,164	£3,514

Table 3 shows the total costs per patient over the 12-month follow-up. While there was no evidence of a significant difference in cost per patient between the RCBT intervention and TAU groups, there was a significant reduction in the mean cost of inpatient hospital use (*P* = 0.031).

Including the cost of providing RCBT, the mean overall cost saving per patient over 12 months with RCBT, healthcare, medication, travel and informal care costs was £1,064 (95% CI –£845–£2973, *P* = 0.269). The mean cost of providing RCBT was calculated at £531.80 (95% CI £466.80–£597.80).

QALYs were 0.66 and 0.59 for the intervention group and the control group, respectively; this difference at 12 months was not significant (Table 2). However, the ICER for QALYs was –£15,200 (the difference in cost, –£1,064, divided by the difference in QALYs, 0·07), which highlights additional expenditure to gain additional QALYs. It is clear to see that the RCBT intervention represents lower cost and more QALYs; that is, the RCBT intervention is in a position of strict dominance.

$$ ICER=\frac{\Delta  Cost}{\Delta  QALY}=\frac{Cost_{CBT}-{Cost}_{TAU}}{QALY_{CBT}-{QALY}_{TAU}}=\frac{\mathit{\pounds}2197-\mathit{\pounds}3261}{0.66-0.59}=\frac{-\mathit{\pounds}\mathrm{1,064}}{0.07}=-\mathit{\pounds}\mathrm{15,200} $$

The uncertainty around the ICER was explored, given that the ICER is based on sample means (Additional file 1: Table S6). A negative ICER is nevertheless not easily interpretable. Therefore, the net monetary benefit (NMB) was calculated as:

$$ \left(\mathrm{incremental}\ \mathrm{benefit}\times \mathrm{willingness}\hbox{-} \mathrm{to}\hbox{-} \mathrm{pay}\ \left(\mathrm{WTP}\right)\ \mathrm{threshold}\right)-\mathrm{incremental}\ \mathrm{cost} $$

A positive NMB indicates that the intervention is cost-effective compared to the alternative and vice versa. We chose a WTP of £30,000, as recommended by NICE guidelines [34]; the NMB at £30,000 is £3,164 with observed values. Positive NMBs were generated using a range of WTPs from £5,000 to £35,000, i.e. both cost-saving and the cost-effectiveness are achieved by the RCBT intervention compared to TAU (Table 4). Overall, the RCBT intervention is in a position of strict dominance, which means delivering the RCBT intervention could reduce costs and demonstrate cost-effectiveness compared to TAU using both the observed and multiple imputation values.

**Fig. 3:**
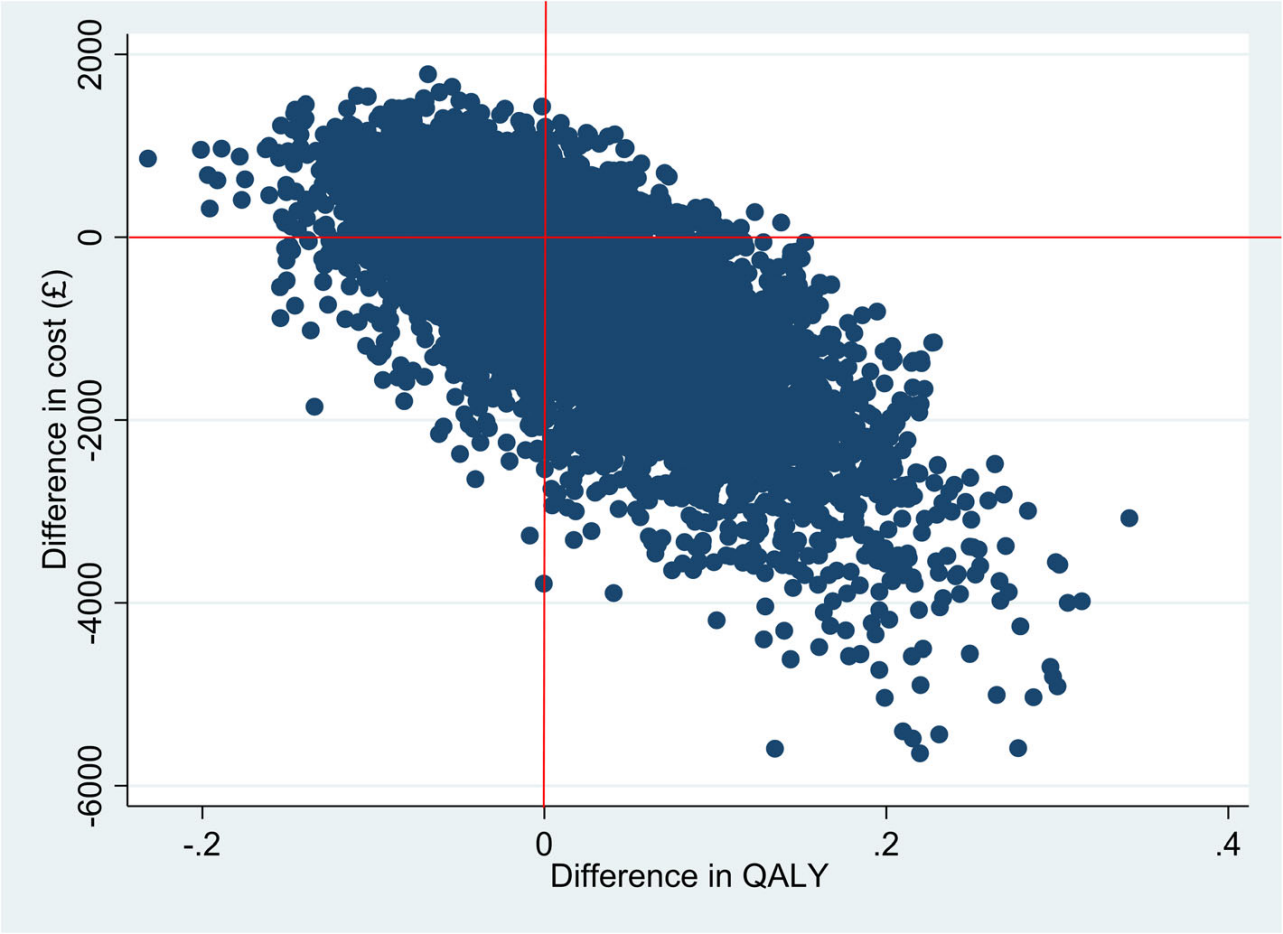
Plot of bootstrapped samples on the cost-effectiveness plan using EQ-5D-5L utilities

Figure 3 illustrates a cost-effectiveness plane (CEP), where the scatter of points is based on the results of a non-parametric bootstrap analysis (5,000 replications) on total costs and QALYs. The scatter of points is mainly based in the southeast quadrant. In the southeast quadrant, the CBT intervention is more effective and less costly for health anxiety and thus dominates TAU. For a given threshold of £30,000 by NICE, all points below this threshold are deemed to be cost-effective because the CBT intervention is dominant in the southeast quadrant.

## Discussion

This study is the first to apply targeted, real-time, remotely delivered CBT to address the clinical needs of repeat users of unscheduled care with severe health anxiety and the related costs to health services. Results suggest that RCBT is effective in reducing severe health anxiety at six months, sustained to at least 12 months, while also reducing comorbid generalised anxiety and depression and improving overall health in patients recruited from primary and secondary care services. The benefits are similar to those achieved when offering face-to-face CBT for health anxiety with more selected groups such as hospital clinic attenders [11, 13]. No differences were found between participants in the RCBT or TAU groups in terms of either somatic symptoms or work and social adjustment. The RCBT intervention showed a position of strict economic dominance, with a net monetary benefit of £3,164 per participant at a NICE-determined WTP threshold of £30,000. Higher rates of recruitment (33% of those eligible) than previous trials of psychological interventions in frequently attending patients with health anxiety [11, 37] suggest that the recruitment methods involving non-stigmatising explanation and RCBT were acceptable. Overall, our results suggest that targeted remote delivery of CBT is a feasible, clinically effective and cost-saving method of improving self-management in repeat users of unscheduled care with severe health anxiety, with whom health systems have previously struggled to engage.

### Strengths

The study successfully recruited participants with high levels of unscheduled healthcare use and severe health anxiety; and those with clinical levels of comorbid depression, generalised anxiety, other bodily symptoms and poor quality of life. The use of telephone and videoconferencing interviews allowed participants of all backgrounds to be engaged and recruited, particularly those who may have been resistant or unable to access traditional mental health services. Most participants were recruited through usual care clinical encounters, particularly in primary care, rather than large-scale screening procedures in general hospital outpatient departments or direct public adverts used in previous studies [9, 38–40]. The methods used may be more easily incorporated into routine health system procedures than has been typical.

This study confirms previous findings that similar treatment effects achieved with face-to-face CBT can also be realised using remotely delivered CBT [11–13]. Furthermore, the pragmatic, applied methods used in this trial mean the results are potentially generalisable to routine clinical practice in UK and other similar health services.

The study further provides evidence that the user-design and co-production approach we applied through networks of practice [23] may have improved both engagement and recruitment to the study as a whole and to RCBT in particular**.** A non-stigmatising explanation of health anxiety and its effects, coupled with the delivery of RCBT, might be utilised as a promising means of engaging repeat users of unscheduled care with severe health anxiety into effective treatment. Furthermore, the higher rates of recruitment might also be attributed to the accessibility and convenience of the RCBT intervention for each participant. The delivery methods also helped provide a complex intervention over a large area, with a comparatively small staff team, in terms of both conducting the research and the RCBT.

### Limitations

The requirement for only two unscheduled care appointments in the previous 12 months may appear arbitrary in triaging patients at high risk of anxiety-related service use. By not setting different thresholds for males and females, we may have recruited a greater proportion of females than males [41]. However, we recruited participants with much higher baseline use of repeat unscheduled care in the preceding year using this easily applied cut-off, suggesting an appropriate set of inclusion criteria for clinical use and that the gender composition of the sample would not have been different if we had applied a higher cut-off score.

We recognise that using digital technology in the intervention group may have led to the selection of younger participants, although participants up to the age of 79 years utilised RCBT. However, use of the internet does not necessarily mean that older participants could not be recruited, nor that RCBT is ineffective in older adults [38–40].

One of the main limitations of the trial was reduced retention in follow-up. However, achieving 72% at six months for our primary outcome is considerable, given the complexities of this patient group, within routine care across sectors. Lower rates of follow-up might be expected in studies of harder-to-engage patients [42]. Similar results were obtained using only participants who provided six months of follow-up data as when using imputed data in the intention-to-treat analysis, suggesting that the results are robust. A further limitation is that lower follow-up rates at three, nine and 12 months than at six months mean there is less precision concerning the treatment effects at these time points than at six months. Further independent replication in a larger sample would be desirable to test the robustness of our findings. We did not inspect patients’ primary care and general hospital records because self-report may be more accurate in patients who frequently utilise multiple sources of unscheduled care from different agencies [37, 43].

Unlike some other internet and face-to-face delivered CBT for health anxiety [15, 38–40], there was no difference in outcome at three months in our RCT. In the current RCT, participants had previously received no diagnosis or explanation for health anxiety, until, that is, health anxiety was explained to all participants as part of the informed consent process. As a result, there was a substantial mean drop in the SHAI score of 6 points from baseline in both treatment groups at three months, with no benefit of RCBT over TAU emerging until six months. Participants commented on the value of receiving an explanation of health anxiety to make sense of their symptoms and, furthermore, around 10% of TAU participants sought psychological treatment over 12 months. In contrast, participants in most previous RCTs already had a diagnosis or understood they might have health anxiety before participation.

### Implications

Further research should explore whether the benefits of RCBT in repeat users of unscheduled care with severe health anxiety are maintained beyond 12 months [11]. Further research is also required to directly compare the parity of effectiveness of RCBT, face-to-face delivery and blended approaches utilising both face-to-face and remote therapies, according to participant and therapist preference.

At a time of financial restraint within health services, this study presents a promising intervention that can both improve the health of a hard-to-reach population and reduce the cost to health services. We will separately report further qualitative data on the acceptability of the intervention. The pragmatic design of this RCT means procedures could be used in routine NHS care to facilitate low-cost implementation. Such an approach requires integration of care: participants are engaged through primary care and some hospital departments that provide continuing care, RCBT is delivered by psychological treatment providers and the benefits to the healthcare system are to the participant, their personal support network (informal care), primary care and hospital services.

For those who are unwilling or unable to engage in face-to-face CBT for health anxiety in existing psychological treatment services, our methods of explanation and remote delivery may provide a cheap, effective and implementable method of delivering CBT to repeat users of unscheduled care with high health anxiety, instead of costly collusion of clinicians in a cycle of repeat investigation, inter-specialist referral, additional medication and clinician perpetuated follow-up [8, 10, 44].

## Conclusion

Compared to usual care, RCBT may reduce health anxiety at six, nine and 12 months, and improve general anxiety, depression and overall health at 12 months. It may also reduce health and informal care costs in the order of £1,000 per patient over 12 months in repeat users of unscheduled care with severe health anxiety. These patients have previously been difficult to engage in psychological treatment, but in this trial, about one-third were successfully engaged.

## Additional file

Additional file 1: **Table S1.** Essentials of specific elements of cognitive behaviour therapy for health anxiety. **Table S2.** Diagnostic and demographic features of participants (N=156). **Figure S1.** Observed mean (95% CI) change in Short Health Anxiety Inventory over 12 months remote cognitive behaviour therapy versus treatment as usual. **Table S3.** Change in SHAI scores over 12 months (observed value). **Table S4.** Recovery and remission rates on 14-item Health Anxiety Inventory in remote cognitive behaviour therapy and treatment as usual groups. **Table S5.** Changes from baseline on SF36 domains in remote cognitive behaviour therapy (RCBT) and treatment as usual (TAU). **Table S6.** Incremental cost effectiveness ratios (ICERS) for remote cognitive behaviour therapy (RCBT) and treatment as usual (TAU). (DOCX 50 kb)

## Abbreviations

CBT: Cognitive behaviour therapy
CHAMP: Cognitive Behavioural Therapy for Health Anxiety in Medical Patients
CSRI: Client Service Receipt Inventory
CTSR: Cognitive Therapy Rating Scale Revised
EQ5D-5L: Euroqol health utility measure
GAD-7: 7-item Generalised Anxiety Disorder scale
ICER: Incremental cost effectiveness ratio
IQR: Interquartile range
NHS: UK National Health Service
NICE: National Institute for Health and Care Excellence
NMB: Net monetary benefit
PHQ-9: 9-item Personal Health Questionnaire
PHQ-15: 15-item Personal Health Questionnaire
PPI: Patient and public involvement
QALY: Quality adjusted life year
RCBT: Remotely delivered cognitive behaviour therapy
RCT: Randomised controlled trial
SF-36: Short Form Health Survey, 36 item version
SHAI: 14-item Short Health Anxiety Inventory
TAU: Treatment as usual
VAS: Visual analogue scale
WASAS: Work and Social Adjustment Scale
WTP: Willingness to pay threshold
