# Supplementary material for: Clinical and economic outcomes of remotely delivered cognitive behaviour therapy versus treatment as usual for repeat unscheduled care users with severe health anxiety: a multicentre randomised controlled trial
Source: BMC Med. 2019 Jan 23;17:16. doi: 10.1186/s12916-019-1253-5 (PMC6343350; doi:10.1186/s12916-019-1253-5)
Supplement: Supplementary file 1 — Table S1. Essentials of specific elements of cognitive behaviour therapy for health anxiety. Table S2. Diagnostic and demographic features of participants (N=156). Figure S1. Observed mean (95% CI) change in Short Health Anxiety Inventory over 12 months remote cognitive behaviour therapy versus treatment as usual. Table S3. Change in SHAI scores over 12 months (observed value). Table S4. Recovery and remission rates on 14-item Health Anxiety Inventory in remote cognitive behaviour therapy and treatment as usual groups. Table S5. Changes from baseline on SF36 domains in remote cognitive behaviour therapy (RCBT) and treatment as usual (TAU). Table S6. Incremental cost effectiveness ratios (ICERS) for remote cognitive behaviour therapy (RCBT) and treatment as usual (TAU). (DOCX 50 kb) [file 12916_2019_1253_MOESM1_ESM.docx]

**Additional file 1: Table S1: Essentials of specific elements of cognitive behaviour therapy for health anxiety**

| - Listing of all symptoms and how the patient interprets them - Formulation of worst fears - Introducing possibility of alternative explanations (e.g. pie charts and pyramids) - Evaluation of risk of disease - Considering the price paid for health anxiety - Exploring the hypothesis of fear of disease being more likely than actual disease - Building evidence by identifying symptom patterns that are related to those of anxiety (i.e. diary keeping) - Reducing behaviour that maintains health anxiety such as excessive bodily checking and reassurance seeking |
| --- |

**Table S2: Diagnostic and demographic features of participants (N = 156)**

|  | RCBT (n = 78), n (%) | TAU (n = 78), n (%) |
| --- | --- | --- |
| Age (years) | | |
| 18–24 | 24 (31) | 25 (32) |
| 25–34 | 20 (26) | 15 (19) |
| 35–44 | 7 (9) | 7 (9) |
| 45–54 | 11 (14) | 9 (12) |
| 55–64 | 8 (10) | 16 (21) |
| 65–74 | 6 (8) | 1 (1) |
| 75 and older | 2 (3) | 5 (6) |
| Ethnicity | | |
| White British | 58 (74) | 58 (74) |
| White Irish | 6 (8) | 3 (4) |
| White Other | 4 (5) | 6 (8) |
| Black/Black British – African origin | 0 | 1 (1) |
| Black/Black British –Caribbean origin | 2 (3) | 3 (4) |
| Asian/Asian British – Indian | 0 | 1 (1) |
| Asian/Asian British – Pakistani | 2 (3) | 1 (1) |
| Asian/Asian British – Chinese | 2 (3) | 1 (1) |
| Arabic | 0 | 2 (3) |
| Mixed – any other mixed | 0 | 1 (1) |
| Mixed White and Black Caribbean | 1 (1) | 0 |
| Any other ethnic group | 3 (4) | 1 (1) |
| Referral site | | |
| Nottinghamshire | 57 (73) | 56 (72) |
| Lincolnshire | 7 (9) | 7 (9) |
| Leicestershire | 1 (1) | 1 (1) |
| Derbyshire | 5 (6) | 6 (8) |
| Northamptonshire | 6 (8) | 5 (6) |
| Stoke-on-Trent | 2 (4) | 3 (4) |
| SCID DSM-V criteria met | | |
| Current major depressive episode | 44 (52) | 37 (47) |
| Current persistent depressive disorder | 21 (27) | 30 (38) |
| Panic disorder | 42 (54) | 49 (63) |
| Agoraphobia | 16 (21) | 15 (19) |
| Social anxiety disorder | 7 (9) | 14 (8) |
| Specific phobia | 8 (10) | 7 (9) |
| Obsessive-compulsive disorder | 15 (19) | 18 (23) |
| Post-traumatic stress disorder | 11 (14) | 11 (14) |
| Current generalised anxiety disorder | 52 (67) | 56 (72) |
| Somatic symptom disorder | 44 (56) | 46 (59) |
| Illness anxiety disorder | 47 (60) | 36 (46) |

**Figure S1.** **Observed mean (95% CI) change in Short Health Anxiety Inventory score over 12 months remote cognitive behaviour therapy versus treatment as usual.**

**Table S3: Change in Short Health Anxiety Inventory scores over 12 months (observed value).**

| **Month** | **RCBT (range)** | **TAU (range)** | **Difference in mean change (95% CI)** | ***P*-value** |
| --- | --- | --- | --- | --- |
| 3 | –5.62 (–7.38 to –3.86) | –5.64 (–7.42 to –3.87) | 0.03 (–2.48 to 2.53) | 0.983 |
| 6 | –9.04 (–10.70 to –7.38) | –6.30 (–7.93 to –4.66) | –2.75 (–4.96 to –0.53) | 0.015 |
| 9 | –9.17 (–10.87 to –7.47) | –6.42 (–8.09 to –4.76) | –2.75 (–4.96 to –0.53) | 0.015 |
| 12 | –10.14 (–11.84 to –8.45) | –7.39 (–9.07 to –5.72) | –2.75 (–4.96 to –0.53) | 0.015 |

**Table S4: Recovery and remission rates on 14-item Short Health Anxiety Inventory (SHAI) in remote cognitive behaviour therapy (RCBT) and treatment as usual (TAU) groups.**

| Time point (months) | SHAI | RCBT (n = 78), n (%) | TAU (n = 78), n (%) |
| --- | --- | --- | --- |
| 0 | ≥15  ≥18  ≥20 | 78 (100)  76 (97)  74 (95) | 78 (100)  78 (100)  67 (86) |
| 3 | ≥15  ≥18  ≥20 | 58 (92)  48 (76)  39 (62) | 49 (77)  45 (70)  38 (59) |
| 6 | ≥15  ≥18  ≥20 | 41 (70)  27 (46)  23 (39) | 50 (82)  41 (67)  32 (52) |
| 9 | ≥15  ≥18  ≥20 | 35 (67)  27 (52)  21 (40) | 41 (76)  37 (27)  27 (50) |
| 12 | ≥15  ≥18  ≥20 | 25 (53)  18 (38)  12 (26) | 34 (72)  27 (57)  19 (40) |

**Table S5: Changes from baseline on SF36 domains in remote cognitive behaviour therapy (RCBT) and treatment as usual (TAU).**

| **Physical function** | **RCBT (range)**  **n = 78** | **TAU (range)**  **n = 78** |  | **Mean change difference (95% CI)** | ***P*-value** |
| --- | --- | --- | --- | --- | --- |
| 6 months | 1.90 (–3.79–7.59) | 4.65 (–1.46–10.76) |  | –2.75 (–10.93–5.42), | 0.508 |
| 12 months | 6.18 (–0.73–13.09) | –1.67 (–8.65–5.32) |  | 7.85 (–1.45–17.15), | 0.097 |
| **Role limitation –physical** | | | | | |
| 6 months | 27.83 (16.70–38.97) | 19.51 (8.95–30.06) |  | 8.33 (–7.14–23.79), | 0.290 |
| 12 months | 22.66 (10.19–35.12) | 22.70 (9.67–35.73) |  | –0.04 (–17.10–17.02), | 0.996 |
| **Role limitation –emotional** | | | | | |
| 6 months | 26.01 (14.01–38.00) | 7.29 (–5.73–20.31) |  | 18.72 (1.06–36.38), | 0.038 |
| 12 months | 28.16 (15.49–40.82) | 18.54 (2.79–34.29) |  | 9.61 (–9.83–29.06), | 0.327 |
| **Emotional well-being** | | | | | |
| 6 months | 15.25 (10.09–20.41) | 13.56 (8.59–18.54) |  | 1.68 (–5.53–8.89), | 0.646 |
| 12 months | 18.41 (12.84–23.99) | 11.38 (5.44–17.32) |  | 7.04 (–1.63–15.70), | 0.110 |
| **Energy/fatigue** | | | | | |
| 6 months | 14.52 (8.44–20.61) | 10.23 (4.91–15.55) |  | 4.30 (–4.26–12.85), | 0.323 |
| 12 months | 14.57 (8.83–20.31) | 10.11 (4.06–16.15) |  | 4.46 (–3.48–12.41), | 0.270 |
| **Pain** | | | | | |
| 6 months | 11.76 (6.08–17.45) | 14.99 (9.42–20.57) |  | –3.23 (–11.40–4.94), | 0.435 |
| 12 months | 13.32 (8.14–18.51) | 11.15 (5.22–17.07) |  | 2.17 (–5.78–10.13), | 0.590 |
| **Social function** | | | | | |
| 6 months | 17.79 (10.83–24.75) | 14.41 (7.26–21.55) |  | 3.38 (–5.95–12.72), | 0.476 |
| 12 months | 23.05 (15.80–30.31) | 17.73 (10.26–25.20) |  | 5.32 (–5.14–15.78), | 0.316 |
| **General health** | | | | | |
| 6 months | 14.48 (9.82–19.13) | 4.76 (0.20, 9.31) |  | 9.72 (3.55, 15.89), | 0.002 |
| 12 months | 14.43 (9.73–19.12) | 6.16 (1.62, 10.70) |  | 8.27 (1.84, 14.70), | 0.012 |

Significant improvements only in role limitations caused by emotional problems at 6 months but not at 12 months and in general health at 6 and 12 months with RCBT versus TAU.

**Table S6: Incremental cost-effectiveness ratios (ICERS) for remote cognitive behaviour therapy (RCBT) and treatment as usual (TAU).**

|  | **RCBT**  **Mean (SD)** | **TAU**  **Mean (SD)** |
| --- | --- | --- |
| QALY at 12 months | 0.66 (0.22) | 0.59 (0.31) |
| Total cost at 12 months | £2197 (1048) | £3261 (5010) |
| ICER | (£2197–£3261)/(0.66–0.59)=–£15,200 | |

QALY: Quality adjusted life year
